# Human papillomavirus-16 presence and physical status in lung carcinomas from Asia

**DOI:** 10.1186/1750-9378-5-20

**Published:** 2010-11-16

**Authors:** Francisco Aguayo, Muhammad Anwar, Chihaya Koriyama, Andres Castillo, Quanfu Sun, Jacob Morewaya, Yoshito Eizuru, Suminori Akiba

**Affiliations:** 1 Molecular Pathology and Epidemiology Laboratory, Public Health Department, Faculty of Medicine, P Universidad Catolica de Chile, Santiago, Chile; 2 Department of Epidemiology and Preventive Medicine, Kagoshima University Graduate School of Medical and Dental Sciences, 8-35-1 Sakuragaoka, Kagoshima, 890-8544, Japan; 3 Division of Individual Monitoring and Health Surveillance, National Institute for Radiological Protection, Chinese Center for Disease Control and Prevention, 2 Xinkang Street, Deshengmenwai, Xicheng District, Beijing 100088, China; 4 School of Medicine and Health Sciences, University of Papua New Guinea; 5 Division of Oncogenic and Persistent Viruses, Center for Chronic Viral Diseases, Kagoshima University Graduate School of Medical and Dental Sciences, 8-35-1 Sakuragaoka, Kagoshima, 890-8544, Japan; 6 Programa de Virología, Instituto de Ciencias Biomédicas (ICBM), Facultad de Medicina, Universidad de Chile, Santiago, Chile

## Abstract

**Background:**

Although human papillomavirus (HPV) genome has been detected in lung cancer, its prevalence is highly variable around the world. Higher frequencies have been reported in far-east Asian countries, when compared with European countries. The present study analysed the HPV-16 presence in 60 lung carcinomas from the Asian countries China, Pakistan and Papua New Guinea.

**Results:**

HPV-16 was present in 8/59 (13%) samples. According to histological type, HPV-16 was detected in 8/18 (44%) squamous cell carcinomas (SQCs), which were mainly from Pakistan; 0/38 (0%) adenocarcinomas (ACs), which were mainly from China; and in 0/4 (0%) small cell carcinomas (SCLCs). The observed histological difference was statistically significant (p < 0.001). HPV-16 viral load was also determined using real-time polymerase chain reaction (qRT-PCR); it ranged between 411 to 2345 copies/100 ng of genomic DNA. HPV-16 genome was found integrated into the host genome in every HPV-16 positive carcinoma.

**Conclusion:**

These results support the notion that HPV-16 infection is highly associated with SQCs in Pakistan. Our results show a frequent HPV-16 integration in SQCs, although the low viral load casts doubt respect a direct etiological role of HPV in lung carcinomas from Asia. Additional HPV-16 characterization is necessary to establish a direct or indirect etiological role of HPV in this malignancy.

## Background

Human papillomaviruses (HPVs) are epitheliotropic double-stranded DNA viruses that belong to the *papillomaviridae *family. Epidemiological, clinical and basic research has allowed to conclude that HPV infection is the necessary cause of cervix uterine cancer [1]. In addition, HPV has been associated with other extra-genital malignancies such as cancers of the head and neck, esophageal, skin and lung [2]. The prevalence of HPV in lung cancer is highly variable around the world. A meta-analysis made by Srinivasan reported that HPV frequency in lung carcinomas ranged between 0 to 78.3% worldwide. Relatively high frequencies are found in Far East Asian countries when compared with European countries [3]. In addition, HPV is suspected to predispose to particular histological subtypes of lung cancer. A study in Okinawa, southern islands of Japan, found that HPV was detected in 79% of the cases, particularly in the well-differentiated SQCs. However, the proportion of HPV-positive lung carcinomas in Okinawa decreased in the 1990 s, in parallel with the falling incidence of well differentiated SQCs, and was only 24% in the late 1990 s [4]. Relatively high HPV prevalence rates in SQCs were found in our study in Chile [5] and studies in central China [6]. However, the association of HPV to lung SQC is not always observed. For example, an Iranian study showed no evident association to any histological types [7]: HPV was found in 25% of SQCs, 21% of ACs, 33% of large cell lung carcinomas and 29% of small cell lung carcinomas (SCLCs). In Europe, one study analysed 218 frozen specimens of lung carcinomas from France, using Roche line blot assay, and found only four HPV positive samples, one poorly differentiated SQC and three large cell carcinomas [8]. A Korean study did not show any histological association of HPV, either [9]. On the other hand, studies conducted in Taiwan and in Kagoshima, Japan, showed a high prevalence of HPV in ACs. In a Taiwanese study, HPV-16 and HPV-18 were positive in 43% and 49% of ACs, respectively, but only in 24% and 29% of SQCs, respectively [10]. Interestingly, a recent study in Kagoshima, Japan, examined 20 additional ACs treated with gefitinib, a tyrosine kinase inhibitor specific for epidermal growth factor receptor (EGFR), and detected high-risk HPV genomes in 75% (7/8) of ACs with complete or partial response to gefitinib but not in the remaining 12, which did not respond to gefitinib [11]. Note that non-smoking women with East Asian ethnicity is a predictive factor of gefitinib responsive ACs [12].

In general, high risk-HPVs are frequently integrated in cervical cancer, and low risk-HPVs are frequently found to be episomal [13]. Moreover, it has been reported that HPV integration is more frequently found in high-grade lesions (invasive carcinoma) when compared with low grade lesions [14]. The HPV integration frequently disrupts E2 ORF causing E6 and E7 overexpression because E2 protein functions as a repressor of p97 promoter in HPV-16. The E6 and E7 overexpression induce p53 and pRb loss, respectively [15]. In cervical carcinogenesis, deregulated expression of these two viral oncogenes in basal cells, mostly by HPV integration, is considered to be a critical event for disease progression [16].

Epidemiological evidence suggests that pathways involved in HPV-induced oncogenesis may be influenced by ethnic factors and anatomical location as well as exposures to risk factors such as smoking and alcohol [17]. Thus, it is of interest to study the prevalence and function of HPV-16 in lung carcinomas from countries with different lifestyles. In the present study we examined lung cancer specimens from Asian countries such as China, Pakistan and Papua New Guinea. Studies on HPV prevalence in lung cancer in Pakistan or Papua New Guinea have not been reported to date. In addition, in order to characterize the role of HPV in this tumour, we determined the physical status (episomal/integrated) and viral load in HPV-16 positive cases.

## Results

The present study analysed the HPV-16 presence in lung carcinomas from Pakistan, China and Papua New Guinea. The clinicopathological features of analysed specimens are shown in Table 1. No statistically significant differences were observed between analysed histological types and age, gender or differentiation status (*P *= 0.295, 0.133 and 0.179, respectively). All ACs analysed in this study were obtained from China and all SQCs were obtained from Pakistan and Papua New Guinea. Thus, in this study there was a statistically significant difference between analysed histological type of lung cancer and countries involved (*P *< 0.05).

**Table 1:** Clinicopathological features of lung carcinomas from China, Pakistan and Papua New Guinea

		All*	histological types			p-value
			ACs	SQCs	SCLCs	
Age						0.295
	-64	38	23 (61)	11 (29)	4 (11)	
	65+	18	14 (78)	4 (22)	0 (0)	
Gender						0.133
	Male	43	24 (56)	16 (37)	3 (7)	
	Female	17	14 (82)	2 (12)	1 (6)	
Differentiation						0.179
	poor	19	11 (58)	8 (42)	0 (0)	
	moderate	26	17 (65)	9 (35)	0 (0)	
	well	11	10 (91)	1 (9)	0 (0)	
Country						< 0.005
	Pakistan	21	4 (19)	13 (62)	4 (19)	
	China	31	31 (100)	0 (0)	0 (0)	
	PNG	8	3 (38)	5 (62)	0 (0)	

* Ages and tumor differentiation was not available in four cases

PNG: Papua New Guinea

**Figure 1:**
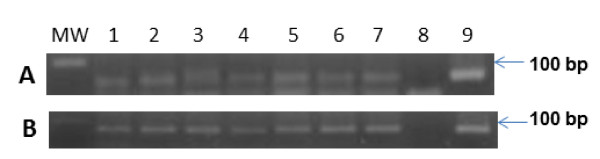
**HPV-16 is detected in lung carcinomas from Asia**. DNA purified from lung carcinomas from Asian patients were amplified by PCR using **A**: generic primers flanking the HPV L1 region (65 bp) and **B**: specific primers flanking the HPV-16 E6 region (96 bp) and revealed by agarose gel electrophoresis and ethidium bromide staining. MW: 100 bp molecular weight marker; wells 1-7: positive clinical samples; wells 8-9: Negative and positive control (DNA from HPV-16 recombinant plasmid).

**Figure 2:**
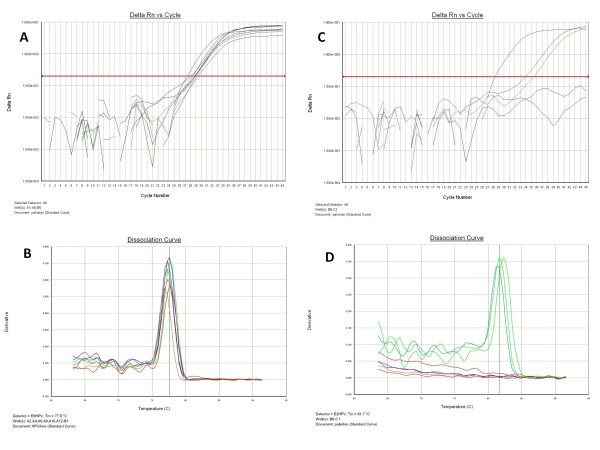
**Quantitative Real-time PCR for HPV-16 in lung carcinomas from Asia**. **A**: Amplification of HPV-16 E6 fragment; **B**: Melting analysis after E6 amplification; **C**: Amplification of HPV-16 E2 fragment; **D**: Melting analysis after E2 amplification.

We successfully purified DNA in all the analysed specimens. The DNA was highly pure (Absorbance 260/280 = 1, 8) and its concentration was ranged between 50 to 100 ng/μL. In addition, to check the DNA fragmentation and presence of amplifiable DNA for PCR, we successfully amplified a 110 betaglobin DNA fragment in all of specimens. PCR with GP5+/6+ primers revealed that 8/60 (13%) cases were HPV positive. In order to determine the HPV genotype, we used PCR for amplification of a HPV-16 E6 specific DNA fragment. All the cases that were HPV GP5+/6+ positive were typed as HPV-16. We confirmed these results using PCR for amplification of another 65 bp DNA fragment of HPV-16 E6 [18] (Figure 1A y 1B). No other HPV genotype was found in this study. The HPV-16 genotype was present in SQCs (8/18) but not in ACs or SCLCs (Table 2) and this difference among histological types was statistically significant (P < 0.001). Gender, age or tumour differentiation was not significantly associated with HPV-16 presence (*P*= 0.091, 0.669 and 0.131, respectively). In addition, we used real-time PCR to determine viral load and physical status of HPV. All the HPV-16 positive specimens were successfully amplified for an 81 bp fragment of E6 gene but only 3 specimens were successfully amplified for an 82 bp fragment of E2 gene (Figures 2A and 2C). The amplified fragments were characterized by melting analysis that showed a specific curve with a Tm (melting temperature) of 77.6°C for the E6 fragment and 81.7°C for the E2 fragment. One specimen positive for E2 gene showed a Tm that was higher 0.5°C approximately compared with the other two specimens (Figures 2B and 2D). The specific presence of amplification products was checked by agarose gel electrophoresis and ethidium bromide staining in all the samples (data not shown). We found that HPV-16 viral load ranged between 411 to 42141 copies/100 ng of genomic DNA, and the HPV-16 genomes were absolutely integrated in 5/8 (63%) positive lung carcinomas. In 3/8 (37%) carcinomas, episomal and integrated HPV genome was present simultaneously (Table 3). No relationship was observed between HPV-16 viral load and integration status.

**Table 2:** Clinicopathological features of HPV-16-positive lung carcinomas

		All N(%)	HPV16 positive N(%)	HPV16 negative N(%)	p-value
Age					0.669
	< 65 years	38	4 (11)	34 (89)	
	> 65 years	18	3 (17)	15 (83)	
Gender					0.091
	Male	43	8 (19)	35 (81)	
	Female	17	0 (0)	17 (100)	
Histology					* < 0.001*
	AC	38	0 (0)	38 (100)	
	SQC	18	8 (44)	10 (56)	
	SCLC	4	0 (0)	4 (100)	
Differentiation					0.131
	Poor	19	5 (26)	14 (74)	
	Moderate	26	3 (12)	23 (88)	
	Well	11	0 (0)	11 (100)	
Country					0.001
	Pakistan	21	7 (33)	14 (67)	
	China	31	0 (0)	31 (100)	
	PNG	8	1 (13)	9 (87)	

**Table 3:** Physical status of HPV-16 in lung carcinomas

Case	E6 Copies/100 ng	E2 Copies/100 ng	E2/E6	Physical status
1	411	0	0	Integrated
2	1737	35	0.02	Mixed
3	2139	0	0	Integrated
4	1724	0	0	Integrated
5	1384	7.6	0.0055	Mixed
6	1876	0	0	Integrated
7	2345	0	0	Integrated
8	42141	21071	0.5	Mixed

## Discussion

In this study HPV-16 was found in 13% of the lung carcinomas. This finding is consistent with other reports where HPV-16 has been the most common HPV genotype detected in lung cancer [3]. HPV-16 genome was detected only in SQCs (44%) but not in the other histological types (Table 2). These results support the notion that SQCs are more strongly associated with high-risk HPV in the areas other than far-east Asia. On the other hand, the absence of HPV genomes in ACs present a contrast to what was found by studies in Taiwan and Japan, where HPV-16 genome was more frequently found in ACs than in SQCs [10,11]. Since ACs responsive to gefitinib tend be located in the lung periphery, tumor location may be an explanation for those discrepant results. Note that ACs surgically resected in the mainland China may be more centrally located.

Epidemiological evidence suggests that pathways involved in HPV-mediated oncogenesis are influenced by ethnic factors and anatomic location as well as exposures to risk factors such as smoking or alcohol [17]. The most important risk factor of lung cancer is smoking. However, no studies reported an evident interactive effect between HPV infection and tobacco smoking on lung cancer risk. On the other hand, a Taiwanese study that showed a high prevalence of high-risk HPV in non-smoking women [10]. Although 14 ACs in our study were mainly from Chinese women, who tend to be non-smokers, we did not have information about smoking habit in these patients. Therefore, we could not examine the possibility of high HPV prevalence in lung ACs among non-smoking women. Another risk among non-smoking Asian women is long exposure to kitchen fumes [19], which is however, not a common exposure in Japan. Recently, a Taiwanese study reported an increased lung cancer risk in relation to mosquito coil use [20], which is commonly used in Asian countries, including Japan. Other possible environmental risk factors may be heavy metals: A Taiwanese study reported the accumulation of chromium and nickels in lung carcinomas [21]. It should also be noted that arsenic poisoning from artesian well water cause black-foot diseases in southern Taiwan, and is suspected to increase the risk of cancers of the lung, bladder and liver [22].

The variable frequencies of HPV-positive lung carcinomas between countries and continents may be explained by differences in sensitivity of the molecular methods used for HPV detection. PCR has been the most popular method used to detect HPV infection [3]; however the different amplification protocols and primers used make it difficult to compare study results.

The present study found HPV-16 integration in cellular genome in every HPV-16 positive carcinoma. Even so, the low HPV load observed in the present study and in our previous study in Chile makes it difficult to interpret its etiological significance. It should be pointed out however that human cancer can be considered as a stem cell disease originated from a small fraction of cancer cells that show self-renewal and pluripotency and are capable of initiating and sustaining tumor growth [23]. HPV may be present only in a small fraction of cancer cells with stem cell like nature present even in advanced tumors. The transmission route of HPV detected in the upper aerodigestive tract cancers is yet unclear. One possible transmission route of HPV to the lung is through the bloodstream as suggested by a few studies [24]. However, it was pointed out that the possibility of bloodstream transmission appears to be low because of the exclusive intraepithelial cycle of life of HPV infection [25]. Moreover, a recent study which compared the second cancer risk of 335 women with invasive cervical cancer and their first degree relatives did not find any increase of lung cancer risk among cervical cancer patients [26]. Another possibility is a sexual transmission. However, considering the fact that Islamic countries have low incidence of cervical cancer [27], which is almost exclusively caused by HPV infection, such a route is unlikely to explain the presence of HPV in SQCs from Pakistan. Since the virus can infect oral mucosa and subsequently, larynx and bronchial tissue, that may be the main source of HPV detected in the lung.

This report has some methodological biases that need to be commented: First, DNA specimens from paraffin embedded tissues are known to be degraded because of heat during embedding and formalin fixation. Therefore, the present study might have underestimated the viral load *"in vivo"*. Second, the amplification through PCR is not able to detect HPV directly in tumoral cells, so in the future a very sensitive "*in situ*" method to detect HPV or an alternative method to obtain only tumoral cells from clinical specimens, as tissue microdissection are warranted.

## Conclusion

The results obtained in this study support the notion that HPV-16 infection is more strongly associated with SQCs than with ACs in the areas other than far-east Asian countries. Even though HPV-16 was frequently integrated in lung carcinomas, its low viral load casts doubt on a direct etiological role, which has been well established in the case of uterine cervical cancer. However, whether HPV-16 has a direct or indirect impact in the tumour development or modulation remains to be investigated.

## Methods

### Study subjects

We examined 60 bronchoscopic biopsy specimens of paraffin-embedded tissue of SQCs (N = 30) and ACs (N = 32) of the lung from Pakistan, China and Papua New Guinea (PNG). The cases from Pakistan (N = 22) were diagnosed in Pathology Department of King Edward Medical University, Mayo Hospital, Lahore, Pakistan from 1996 to 2002. Cases from PNG were diagnosed in the period 1986-88 at Pathology Department of Port Moresby General Hospital, the major teaching center of School of Medicine & Health Sciences, University of Papua New Guinea. Cases from China were diagnosed during the period between 1989 and 2002 in hospitals in eastern China. Based on 1982 WHO classifications of lung tumors, lung carcinomas were classified into four broad categories: SQCs, ACs, small cell carcinomas and large cell carcinomas [28]. In the present study, histological classification was made using the guideline of the Japan Lung Cancer Society [29], which follows the WHO classification. The institutional review board of the Kagoshima University Graduate School of Medical and Dental Sciences of Japan approved the present study.

### Polymerase chain reaction (PCR) and Southern blotting

Ten-μm-thick sections of each formalin-fixed paraffin-embedded sample were prepared. The samples were added with 1 mL of xylene, and then with 1 mL of ethanol. After centrifugation, the pellet was resuspended in digestion buffer (50 mM Tris-Cl pH 8.0, 1 mM EDTA pH 8.0, 0.5% Tween 20) containing 200 μg of Proteinase K (Invitrogen Corp., USA) and incubated during 24 hours at 56°C. The solution was heated at 100°C for 10 min and centrifuged at 10,000 rpm for 10 min. After phenol/Chloroform extraction, the obtained DNA was quantified using the Nanodrop 1000 spectrophotometer. HPV amplification with GP5+/GP6+ primers [30] was made in a reaction mixture that contained 2.5 μL of sample (containing 100 ng of DNA), 200 μM dNTP, 0.5 μM of each primer and 1.0 U Taq DNA polymerase (Takara, Japan) in a total volume of 25 μL of reaction buffer (50 mM KCl, 20 mM Tris-Cl, pH 8.3). A "hot start" protocol was used with the following conditions of amplification: initial denaturation at 95° for 4 min; subsequent 45 cycles at 95°C for 1 min, 40°C for 2 min and 72°C for 1.5 min and final extension at 72°C for 5 min. The amplification of a betaglobin fragment with a set of PCO3/PCO4 primers (Takara, Japan) was used as internal positive control. All the analysed samples were betaglobin positive. The PCR program was as follows: initial denaturation at 95°C for 4 min, 40 cycles with the cycling profile of 95°C for 1 min, 52°C for 1 min and 72°C for 2 min and final extension for 5 min at 72°C. In addition, the generic HPV presence was detected using another L1 region as previously reported [31]. The specific HPV-16 amplification was made according to the following conditions: initial denaturation at 95°C for 5 min, 45 cycles with the cycling profile of 94°C for 45 s, 55°C for 45 s and 72°C for 45 s and a final extension for 5 min at 72°C. The amplified products were detected through electrophoresis on 3.0% agarose gel.

### Quantitative Real-time PCR

Real-time PCR was performed using the ABI Prism 7000 Sequence Detection System and SYBR-Green PCR master mix (PE, Applied Biosystems, USA). The amplification conditions were 10 min at 95°C and a two-step cycle of 95°C for 15 sec and 60°C for 60 sec for a total of 45 cycles. The primers used were: E6F: gagaaactgcaatgtttcaggacc and E6R: tgtatagttgtttgcagctctgtgc, E2F: aacgaagtatcctctcctgaaattattag and E2R: ccaaggcgacggctttg that amplify a fragment of 81 and 76 bp of E6 and E2 ORFs, respectively [32]. The final concentration of the primers was 0.5 μM. Two standard curves for the E2 and E6 fragments were made by amplification of dilutions between 1 × 10^7 ^and 1 × 10^1 ^copies of HPV-16 cloned in pUC plasmid (kindly given by Dr. Massimo Tommasino, IARC, France). The specificity of amplification was confirmed using dissociation analysis starting at 60°C and agarose gel electrophoresis of the amplified products. In order to find out the physical status of HPV-16, the ratio of E2 to E6 copy numbers was calculated.

### Statistical analysis

Fisher's exact test was used to examine the statistical significance of the results. A p-value <0.05 was considered statistically significant. P values presented are two-sided.

## Competing interests

The authors declare that they have no competing interests.

## Authors' contributions

FA, SA and CK conceived of the study, analysed the data and participated in the redaction of the manuscript. FA and AC made all the analysis of the clinical specimens. MA, QS and JM gave clinical information and got specimens for analysis. YE and CK gave analytical support for all the analysis. All authors read and approved the final manuscript.
